# A review of transition experiences in perinatally and behaviourally acquired HIV-1 infection; same, same but different?

**DOI:** 10.7448/IAS.20.4.21506

**Published:** 2017-05-16

**Authors:** Phung Khanh Lam, Sarah Fidler, Caroline Foster

**Affiliations:** ^a^ Department of Infectious Diseases, Oxford University Clinical Research Unit, Ho Chi Minh City, Vietnam; ^b^ 900 Clinic, Department of HIV and GU Medicine, Imperial College Healthcare NHS Trust, London, UK

**Keywords:** Transition experience, HIV, adolescents, young people, modes of infection

## Abstract

**Introduction:** Despite sharing common psychosocial and developmental experiences, adolescents living with perinatally and behaviourally acquired HIV-1 infection are different in terms of timing of HIV infection and developmental stage at infection. Therefore, it is of interest to identify similarities and differences between these two groups of adolescents living with HIV in their experiences, facilitators and barriers during the transition process.

**Methods:** A detailed literature search of peer-reviewed published papers was conducted on PubMed to identify relevant original research or viewpoints published up to September 2016. Conference abstracts and other unpublished data sources were not included.

**Results:** Existing published literature, mainly using qualitative methods, explores the transition from paediatric to adult healthcare provision, as experienced by these two groups of young people. Reports highlight the variation and similarities in their experiences and challenges of transition. Findings from the USA and Europe predominate, while experience from Africa and Asia is lacking, despite the importance of these regions in the global epidemic.

**Conclusions:** Published transition data remain limited, and there are few studies focusing on behaviourally infected adolescents and key population groups (e.g. adolescents who use drugs, lesbian/gay/transgender individuals). Robust definitions of the transition process and standardized outcome measures are required to facilitate cross-study and geographic comparisons.

## Introduction

Recent estimates [1,2] suggest that there were 1.8 million adolescents living with HIV in 2015, with 250,000 new infections in this group. The ratio between perinatal and behavioural infections is generally unknown [3]. However, a UNAIDS estimate in 2013 suggested that among adolescents living with HIV in the 25 countries contributing the majority of AIDS-related deaths among this age group, around half were infected through mother-to-child transmission, and half through sexual and unsafe injection transmission [4].

In higher-resourced settings, adolescents with perinatally acquired HIV are usually seen by paediatric healthcare providers, and by the time of transition of care to adult services are more likely, when compared to their behaviourally infected peers, to have advanced disease, physical and neurocognitive deficits, HIV-1-associated drug resistance, and drug side effects [5–11]. Global guidelines recommend commencing ART in all age groups [12,13] and hence many perinatally infected children are highly treatment experienced as they enter adolescence. There has been increasing recognition of the emergence of resistance mutations to first-line therapy in resourced-limited settings where access to viral load monitoring and to second-line regimens remains challenging [14,15].

In contrast, adolescents with behaviourally acquired HIV may be seen from diagnosis in adult services, particularly where adolescent services are poorly developed and paediatric care ceases, depending on the setting, between 12 and 18 years of age. As such, they will not experience transition of healthcare provision from paediatric to adult services; yet, they too will be experiencing multiple transitions from childhood to adulthood during this time. Exposure groups at greater risk of HIV acquisition during adolescence include young heterosexual women, men who have sex with men (MSM), transgender adolescents, and those who sell sex and use drugs [16–19]. Risk factors for HIV acquisition in adolescents include: increased number of sexual partners, younger age of sexual debut, previous STIs, mental health diagnosis, poverty, homelessness, and drug and alcohol use [16–19]. As they enter adult care, behaviourally infected adolescents are more likely to be in early stages of disease, the majority with normal prior physical and cognitive development, requiring simpler first-line treatment regimens. However, they may continue to engage in high-risk behaviours and may have difficulties in accepting their diagnosis and treatment [5–7].

In addition to the shared challenges due to the psychosocial and developmental changes of adolescence and the stigma associated with HIV infection, adolescents in both groups frequently lack adequate emotional support, including from family members. This stems in part from difficulty in disclosing their HIV status to relatives or friends, and in the perinatally infected group because many have lost one or both parents to HIV [5–7,20]. Furthermore, adolescents in key populations may have to confront the additional stigma and discrimination related to their drug use or sexual orientation not only among individual healthcare providers, but also within healthcare systems and policy levels [1,21].

This narrative literature review describes key differences and similarities in transition experiences between populations of HIV-positive youth by mode of infection, what is known about their outcomes in adult care, and future research priorities.

## Methods

We searched PubMed for published English language literature on adolescent transition up to September 2016 using the following search terms: “Adolescent” AND “HIV” AND “Transition”). Of 270 citations retrieved, we focused on any study exploring facilitators and barriers of the transition process among adolescents or young adults living with HIV, and among healthcare workers. We paid particular attention to findings reported by mode of transmission, and we excluded papers which provided only quantitative outcomes in one risk group.

## Results

We identified 25 relevant articles describing the transition experience of adolescents/young adults living with HIV [5,22–45]. While covering different global settings, almost all were from well-resourced regions, where early access to antiretroviral therapy (ART) has resulted in establishment of dedicated transition programmes as adolescents move from paediatric to adult services (Table 1). There were five articles from resource-limited countries (one from Brazil, two from Thailand and two from Africa [28,34–36,44], with most of the remainder from USA and Europe. Data on transition experiences were difficult to compare between settings and even within individual countries, due to the wide variation both in transition services and age at transition, ranging from 12 to 24 years. The majority of the articles described findings from qualitative studies (19/25, 76%), where expectations and experiences of adolescents [22–26,28–35,37], guardians [22,25,26,35], service providers [22,29,34,35,41–45], and policy-makers [35] were assessed. Of the 20 studies assessing transition expectations and experiences of adolescents, 8 focused on adolescents with perinatal infection [22–26,28,29,46], two focused on adolescents with behavioural infection [30,31], and 10 recruited a mixture of adolescents with different types of HIV exposures [5,32–40]. Two common models of transitional care described in these studies were [1] integrating transition services into paediatric or adult clinics [27,32,36], and [2] designing and utilizing a special adolescent clinic that can facilitate transition from paediatric to adult healthcare provider [24,28,38,40]. None of the studies directly compared these models. Few studies evaluated quantitative outcomes post-transition across different HIV exposure groups, and all had very small sample sizes, limiting further interpretation [38–40].

**Table 1.** Summary of published studies describing the transition experience of adolescents living with HIV.

			Reported facilitators	Reported barriers
Study, year, country	Method	Participants	*or* Quantitative outcomes comparing risk groups	
**Perinatal infection**				
Vijayan 2009 [22] USA	Qualitative	18 PHIV, 15 guardians, 9 paediatric providers		Negative perceptions of stigma Lack of autonomy Strong attachment with paediatric provider Negative perceptions of adult provider
Campbell 2010 [23] UK	Qualitative	6 PHIV	Sense of independence Incentives Activities Peer support Educational support, especially on medication	Disclosure
Bundock 2011 [24] UK, Australia	Quantitative Qualitative	21 UK PHIV, 39 AUS young adult with diabetes	Adult provider who is open-minded, receptive and respectful Age-appropriate environment Non-stigmatizing environment Communication between providers and young people Positive relationship with staff Transitioning when ready Well-prepared transition process Sense of independence and self-control	Lack of understanding of adolescent’s needs
Fair 2012 [25] USA	Qualitative	40 PHIV, 18 guardians	Well-prepared transition process Sense of independence and self-control	Strong attachment with paediatric provider Discontinuity of care Lack of preparation for transition
Sharma 2014 [26] USA	Qualitative	15 PHIV, 8 guardians	Personal responsibility Comprehensive care model	Difference between paediatric and adult providers Lack of financial support in adult care Lack of preparation for transition Privacy/Disclosure Negative perceptions of adult provider Paediatric providers too protectively
Righetti 2015 [27] Italy	Quantitative	45 PHIV	84% were retained in care 10 years from the beginning of the transition process. 96% required personalized psychotherapeutic programs, mostly related to HIV diagnosis disclosure. After transition, 98% had personalized antiretroviral therapy, 98% were involved in health education activities and 73% were involved in sexual education activities	
Machado 2016 [28] Brazil	Qualitative	16 PHIV	Pre-connection with the adult team	Lack of preparation for transition Strong attachment with paediatric provider Psychosocial issues Negative perceptions of adult provider
Newman 2016 [29] Australia	Qualitative	12 PHIV and 12 clinicians	Focusing on what young people can gain from becoming independent rather on what they will lose Clinician’s skills to engage young people whilst accepting they are responsible for managing their own wellbeing	
**Behavioural infection**				
Valenzuela 2011 [30] USA	Qualitative	10 BHIV	Well-prepared transition process Options and control in the process Assistance with coordination and linking of services HIV and additional services in the same place Sense of independence and self-control Positive relationship with adult providers	Strong attachment with paediatric provider Lack of preparation for transition Negative perceptions of adult provider Differences between paediatric and adult providers Lack of financial support Privacy/Disclosure Lack of additional services
Hussen 2015 [31] USA	Qualitative	20 BHIV	Individual’s resilience Strong support network Comprehensive support including mental health and education	Level of physical illness at the time of HIV diagnosis Age and developmental stage at HIV diagnosis
**Both modes or unspecified**				
Miles 2004 [32] UK	Qualitative	3 BHIV and 4 PHIV	Adult care-provider integration Visit to adult services Sense of independence and self-control	Strong attachment with paediatric provider Differences between paediatric and adult providers
Maturo 2011 [5] USA	Viewpoint	BHIV and PHIV	Well-prepared transition process Multidisciplinary team	Lack of support from adult provider Psychosocial issues Lack of financial support in adult care
Wiener 2011 [33] USA	Qualitative Quantitative	10 transfusion, 1 BHIV, 48 PHIV	Maintain continuity of care Same doctor at every visit Assistance with the logistical aspects Inclusion of primary caregivers in decision-making and treatment planning Given more responsibility while still in paediatric care	Lack of preparation for transition Lack of financial support Lack of communication between the paediatric and adult providers Lack of consideration of adolescent’s developmental level and competencies Lack of additional support services Negative perceptions of adult provider Differences between paediatric and adult providers
Pettitt 2013 [34] Multicountry, sub-Saharan Africa	Qualitative	8 YHIV, 26 programme managers/service providers	Including YPHIV in transition planning	Lack of preparation for transition Strong attachment with paediatric provider Lack of financial support Lack of additional support (food)
Tulloch 2014 [35] Thailand	Qualitative	6 YHIV, 20 policy-makers, 29 caregivers, 10 prior caregivers, 3 providers	Peer support Life-skills camp	Strong attachment with paediatric provider Psychosocial issues Lack of capacity in adult services Lack of special provision of targeted services for adolescents
Hansudewechakul 2015 [36] Thailand	Viewpoint	Providers’ viewpoint on YHIV	Transitioning youth in groups Youth attending adult care together Peer support Individual readiness to transition Involvement of paediatric and adult healthcare providers	
Kronschnabel 2016 [37] USA	Qualitative	20 YHIV		Lack of preparation for transition Strong attachment with paediatric provider Negative perceptions of adult provider Lack of responsibility and skill development Privacy/Disclosure Lack of social support
Maturo 2015 [38] USA	Quantitative	34 BHIV, 4 PHIV	Non-completion of the transition process was not associated with prevalence of adherence issues, substance use, mental health or pregnancy/childrearing	
Ryscavage 2016 [39] USA	Quantitative	31 BHIV, 19 PHIV	Overall 50% were retained in care 12 months post-linkage. BHIV transferred at older age than PHIV. Linkage and retention in adult care did not differ by exposure group. CD4 and viral load did not differ pre- versus post-transition	
Westling 2016 [40] Sweden	Quantitative	3 BHIV, 31 PHIV	Post-transition, virtually all had VL<50c/mL despite resistance problems and complex social factors. Multidisciplinary approach thought to contribute to good treatment outcomes	
**Medical providers**				
Fair 2010 [41] USA	Qualitative	19 medical providers and social workers	Well-prepared transition process Maintain continuity of care Sense of independence and self-control Assessing readiness for transition	Differences between paediatric and adult providers Psychosocial issues Lack of support on housing and transportation Lack of network of social services Lack of financial support
Gilliam 2011 [42] USA	Qualitative	Staff from ATN clinic sites	Ability and motivation to function independently Strong social support system Financial support Transportation and housing support	Lack of financial support Lack of tracking system Psychosocial issues Stigma Difference between paediatric and adult providers
Newman 2014 [43] Australia	Qualitative	12 paediatric and adult clinicians	Formal transition process Well-prepared transition process Maintain continuity of care Adult provider who can have open conversations with young PLHIV about issues around disclosure, risk behaviours, and sexuality Peer support Psychosocial support	Psychosocial issues Lack of preparation for transition Stigma Lack of communication between the paediatric and adult providers Lack of financial support Lack of transportation support Difference between paediatric and adult providers Lack of tracking system
Kung 2016 [44] South Africa	Qualitative Quantitative	07 healthcare providers (interview) 43 healthcare providers (survey)	Peer support Formal transition process Comprehensive support including sexual health, primary care, dental care, on-site pharmacy services Adult care-provider integration	Lack of a structured healthcare transition Lack of communication between the paediatric and adult providers Differences between paediatric and adult providers Mental health problems Strong attachment with paediatric provider Privacy/Disclosure Negative perceptions of adult provider Stigma
Tanner 2016 [45] USA	Qualitative	174 interviews with clinic staffs		Differences between paediatric and adult providers Psychosocial issues Lack of financial support

BHIV, behaviourally infected; PHIV, perinatally infected; YHIV, young people living with HIV.

Multiple barriers and facilitators to successful transition were identified in the research studies. Amongst identified barriers, the strong attachment between adolescents and the paediatric provider was the most commonly identified factor [22,25,28,30,32,34,35,37,44], which applied to both perinatally and behaviourally infected adolescents. This was reported by adolescents themselves who expressed not wanting to leave their paediatric team, and by the healthcare professionals who cited concerns regarding the adolescents’ ability to self-manage within the healthcare environment of adult services [20,21,23–25,32,33]. This attachment has been explained by the positive long-term relationship between adolescents and providers, the protective care of paediatric providers [26], and the lack of preparation for transition within the healthcare system (e.g. requiring provider time) and for the patient (e.g. knowledge and skills to live independently) [25,26,28,30,33,37,43]. When transition occurred despite sufficient preparation, this attachment led to feelings of loss [25], with some adolescents finding it more difficult to build good relationships with the adult provider.

However, a comprehensive transition programme that was centred around the adolescent and included a formal written policy, time for education and skill development (especially around disease knowledge, scheduling of appointments and medication adherence), and a readiness assessment, was reported to be a facilitator of the transition process [5,24,25,27,30,33,34,41,43,44]. Transition programmes that focussed on helping adolescents build their competence and independence further seemed to improve transition outcomes [23–25,30,32,41,42]. A common theme was for adolescents to have some control over the transition process, and for it to be geared to the adolescent’s individual readiness to transition.

A commonly cited barrier was the negative perceptions among both groups of adolescents and their guardians about adult healthcare providers [22,26,28,30,33,37]. These arose from differences between adult and paediatric services in terms of patient load, expectation of patient autonomy, length and frequency of appointments, clinic setting and patient population [26,30,32,33,41–43,45]. Developing good relationships with adult providers to facilitate transition has been achieved in some settings by integrating adult healthcare professionals into the paediatric clinic, arranging for adolescents to visit the adult provider before transfer, and seeing the same adult clinician each visit [24,28–30,32,33,43,44]. Education for adult healthcare providers around the needs of adolescents, including communication styles and awareness of the potential neurocognitive impact of perinatally acquired HIV and the impact on autonomy, self-care and information processing, further supported the transition process [24,28–30,32,33,43,44].

Another important issue were the psychosocial challenges faced by all adolescents that are further complicated by living with HIV. Hence, papers highlighted how adolescents needed a comprehensive integrated package of support from healthcare providers encompassing mental health [5,27,31,35], sexual and reproductive health [43], substance abuse services and social support integrated within their HIV care [30,33,37]. Whilst paediatric services were typically multidisciplinary in structure, adult services were sometimes “fragmented”, with limited resources, skills and experience in dealing with the complex and varied psychosocial needs of adolescents living with HIV [24,33]. Multidisciplinary support in adult care, especially for mental health, may improve continuity of care and therefore could facilitate the transition process [27,33,41,43]. In young men who have sex with men, an individual’s resilience or coping ability was affected by the strength of their immediate surrounding support network, and important factors influencing successful transition included the level of physical illness, age and developmental stage at the time of their HIV [31].

Confidentiality and fears concerning onward disclosure were highlighted as important barriers to the transition process. Due to their negative perception and/or experience of HIV-associated stigma, adolescents expressed concern about privacy in adult services [26,30,37,42–44]. Adolescents with behavioural infection were particularly concerned about onward disclosure of their HIV status to their families [30], which could lead to poor family support around the transition period. In contrast, perinatally infected adolescents frequently had to negotiate transition alone due to being orphans, creating a very difficult setting for transition compared to other chronic diseases of childhood where the parents have been shown to be key facilitators in the process [47]. In addition, for many perinatally infected adolescents, adult services were often offered within a sexual health setting, before they had become sexually active, which created discomfort and was a regular reminder that they were living with a sexually transmissible infection. System-level issues including lack of social support for housing, transportation and financial resources also negatively affected the transition process and were cited as common barriers to remaining in care [30,33,34,41–43,45].

## Discussion and conclusions

This narrative review of published studies highlights the commonalities in transition experiences for adolescents with perinatally and behaviourally acquired HIV, and the multitude of factors which may be associated with positive and negative transition outcomes in these groups. Common barriers to transition included strong attachments between adolescents and paediatric providers, and negative perceptions of adult healthcare providers. Positive facilitators included having clear policies around transition, education and skills development for providers and patients, assessment of readiness to transition, shared appointments between paediatric and adult providers, and specific training for adult providers on communicating with adolescents. Psychosocial challenges, including stigma, levels of family support, and mental health, were varied and differed by population group, as did the need for different aspects of multidisciplinary care packages.

The studies reviewed highlight the paucity of published data on HIV-specific transition, and particularly studies focusing on behaviourally infected adolescents. Additionally, the lack of standard definitions/endpoints for assessing successful transition complicates interpretation of existing data. This applies to both quantitative (retention, CD4 count, viral load, HIV-1 associated resistance mutations, AIDS and non-AIDS mortality, morbidity) and qualitative (patient and healthcare provider experience, autonomy and self-care) studies. Tracking transition outcomes is further complicated by the lack of robust systems that monitor patients after transfer to adult care [42,43].

Most published findings were from higher resourced settings (mainly the USA and Europe), while data from Africa and Asia are lacking and of particular importance, as these regions have the largest burden of HIV disease with limited resources. Furthermore, there is a need to investigate the impact of cultural differences and the experiences of key population groups on transition outcomes. Understanding the expectations and experiences of adolescents and young adults as they go through transitional care will provide important knowledge to improve current practice.

The priority medical and psychosocial outcomes following any transitional care model are to ensure retention in HIV care, facilitate long-term self-care, and to provide ongoing holistic support to promote health and wellbeing. Young people living with HIV need systems they can trust that offer them open access and understand the need for dialogue around the specific challenges they face. If we are to achieve the UNAIDS global targets of zero AIDS deaths and zero new infections, adolescents and young adults must be respectfully and appropriately engaged in care that is tailored to their complex needs. Patterns of healthcare and health-seeking behaviours established in adolescence can form a framework for lifelong service utilization, and should be nurtured and guided as we seek to help them transition to adult life.
